# Supplementary material for: Acceptability and implementation potential of colorectal cancer screening and health literacy training: A qualitative study among general practitioners in deprived areas
Source: PLoS One. 2025 Feb 11;20(2):e0317910. doi: 10.1371/journal.pone.0317910 (PMC11813080; doi:10.1371/journal.pone.0317910)
Supplement: S2 Appendix — (DOCX) [file pone.0317910.s002.docx]

**DECODE interview guides**

*Interview guide for GPs (up to date from 5^th^ June 2023)*

**1. What do you think of online training?**

- What did you get out of it?

- What did you like about it?

- What did you not like? Why or why not?

- What would you change?

**2. (if relevant) What do you think of the refresher course after 6 months?**

**3. (if relevant) What did your colleagues who took part in DECODE think of the online training?**

- What do the other members of your team (nurse, nurses, interns) think?

**4. On a scale of 0 to 10, to what extent would you recommend the online training to another GP, with 0 being "completely inadvisable" and 10 being "strongly recommended"?**

- Why or why not?

**5. What do you think about a national roll-out of the training to all GPs in France?**

- Why or why not?

- How could we distribute it?

- Do you have any concerns about national distribution?

**6. Do you have any suggestions for improving online training?**

**7. What do you think is the purpose of the brochure and video shown to patients?**

**8. What do you think of the brochure and video?**

- What stands out?

- What did you not like about it? Why or why not?

- What did you like about it? Why or why not?

**9. What did your patients think of the brochure and video?**

- What did the other members of your team think?

**10. Could you briefly describe how you used the brochure and video with eligible people?**

- Does it take up too much time?

- Does it disrupt your consultation?

**11. On a scale of 0 to 10, to what extent would you recommend the intervention for patients, with 0 being "completely inadvisable" and 10 being "strongly recommended"?**

- Why or why not?

**12. What do you think of the routine use of brochures and videos for patients in France?**

- Why or why not?

- Do you have any concerns about their use with your patients?

**13. On a scale of 0 to 10, how likely are you to use the brochure and video with your patients now that the study is over, with 0 being "completely unlikely" and 10 being "completely likely"?**

- Why or why not?

- If the score is greater than 5: would this change your practice?

- If so, how?

**14. For your place of practice, what does the ideal process for using the brochure and video systematically look like?**

- Can you identify any specific actions or procedures that would be required?

**15. Do you have any suggestions for improving the brochure and video?**

*Before ending the interview, I would like to ask you a few final questions about your participation in the DECODE study.*

**16. Why did you agree to take part in a randomised trial like DECODE?**

- Were you motivated by the relevance of the research?

- Were you motivated by the prospect of training?

- Were you motivated by the remuneration?

- Does the subject of the study relate to a problem that you frequently encounter in your clinical practice?

**17. What facilitated your participation in the DECODE study?**

- What were the obstacles to your participation?

**18. To what extent have the DECODE study procedures been integrated into your clinical practice?**

- On the contrary, did the DECODE procedures interfere with your clinical practice?

- Which DECODE tasks were the most complicated to implement?

**19. What do you think of the role of general practitioners in research?**

**20. Do you think that participation in the DECODE study will influence your participation in other studies?**

- Can you explain why?

**21. Do you have any questions or anything else to add?**

*Interview guide for patients (up to date 20^th^ January 2023)*

**1. When and how did you receive the brochure and video?**

- Did you have enough time to watch them?

- Would you have liked to receive the brochure and video earlier?

- By post before your visit to the doctor?

- Or after seeing your doctor?

**2. What do you think of the brochure?**

- What do you think of the text?

- What do you think of the images?

- What did you dislike?

- What did you like?

**3. On a scale of 0 to 10, how would you describe the difficulty of understanding the content of the brochure, 0 being the least difficult and 10 the most difficult?**

- Why or why not?

- Were any words difficult to understand? Which words?

- Were any of the images difficult to understand?

**4. What did you think of the video?**

- What didn't you like?

- What did you like?

**5. Have you shown the brochure or video to your friends and family?**

- If so, what was their impression?

**6. On a scale of 0 to 10, how likely would you be to recommend the brochure and video to someone close to you, with 0 being "completely inadvisable" and 10 being "strongly recommended"?**

- Why or why not?

**7. Do you think the brochure and video should be used systematically in France?**

- Why or why not?

- Do you have any concerns about their use with other patients?

- Do you think the brochure and video are accessible/intelligible to the majority of people concerned?

**8. Do you have any suggestions for improving the brochure?**

**9. Do you have any suggestions for improving the video?**

**10. Do you have any questions or anything else to add?**
