# Supplementary material for: Acceptability and implementation potential of colorectal cancer screening and health literacy training: A qualitative study among general practitioners in deprived areas
Source: PLoS One. 2025 Feb 11;20(2):e0317910. doi: 10.1371/journal.pone.0317910 (PMC11813080; doi:10.1371/journal.pone.0317910)
Supplement: Attachment — Submitted filename: Response to Reviewers.docx [file pone.0317910.s003.docx]

**Responses to academic editor and reviewers**

We thank the editor and reviewers for their valuable feedback

Academic editor’s comments:

**1. When submitting your revision, we need you to address these additional requirements.**

**Please ensure that your manuscript meets PLOS ONE's style requirements, including those for file naming. The PLOS ONE style templates can be found at**

https://journals.plos.org/plosone/s/file?id=wjVg/PLOSOne_formatting_sample_main_body.pdf and

<https://journals.plos.org/plosone/s/file?id=ba62/PLOSOne_formatting_sample_title_authors_affiliations.pdf>

**Answer:** Regarding the affiliations, the SESSTIM has its own publication charter (starting with AIX-Marseille Univ). Please let us know how to proceed. For more information, feel free to contact the trial manager Dr Niamh Redmond [niamh-maria.redmond@univ-tlse3.fr](mailto:niamh-maria.redmond@univ-tlse3.fr)

**2. We note that the grant information you provided in the ‘Funding Information’ and ‘Financial Disclosure’ sections do not match.** When you resubmit, please ensure that you provide the correct grant numbers for the awards you received for your study in the ‘Funding Information’ section.

**Answer:** Upon resubmission, we aimed to include all the required information in both the Financial Disclosure and under “Funding Information» sections but we experienced difficulty identifying where exactly these two sections are located. Here is the correct information in response to your comment:

“This research was supported by a grant (2020-006) from the French National Institute of Cancer (INCa) <https://en.e-cancer.fr/> awarded to **MAD and AL**. The funders had no role in study design, data collection and analysis, decision to publish, or preparation of the manuscript.

Please let us know if there are any further issues with this.

**3. Thank you for stating the following in the Competing Interests section:** "I have read the journal's policy and one author of this manuscript have the following competing interests: MA-D has contributed to the development of Option Grid patient decision aids. EBSCO Information Cervices sells subscription access to Option Grid patient decision aids. She receives consulting income from EBSCO Health, and royalties. No other competing interests declared."

Please confirm that this does not alter your adherence to all PLOS ONE policies on sharing data and materials, by including the following statement: "This does not alter our adherence to PLOS ONE policies on sharing data and materials.” (as detailed online in our guide for authors http://journals.plos.org/plosone/s/competing-interests). If there are restrictions on sharing of data and/or materials, please state these. Please note that we cannot proceed with consideration of your article until this information has been declared.

Please include your updated Competing Interests statement in your cover letter; we will change the online submission form on your behalf.

**Answer:** The following statement: “This does not alter our adherence to PLOS ONE policies on sharing data and materials” was included both in the cover letter and at the end of manuscript.

**4. We note that you have indicated that there are restrictions to data sharing for this study.** For studies involving human research participant data or other sensitive data, we encourage authors to share de-identified or anonymized data. However, when data cannot be publicly shared for ethical reasons, we allow authors to make their data sets available upon request.

For information on unacceptable data access restrictions, please see http://journals.plos.org/plosone/s/data-availability#loc-unacceptable-data-access-restrictions.

Before we proceed with your manuscript, please address the following prompts:

a) If there are ethical or legal restrictions on sharing a de-identified data set, please explain them in detail (e.g., data contain potentially identifying or sensitive patient information, data are owned by a third-party organization, etc.) and who has imposed them (e.g., a Research Ethics Committee or Institutional Review Board, etc.). Please also provide contact information for a data access committee, ethics committee, or other institutional body to which data requests may be sent.

b) If there are no restrictions, please upload the minimal anonymized data set necessary to replicate your study findings to a stable, public repository and provide us with the relevant URLs, DOIs, or accession numbers. Please see http://www.bmj.com/content/340/bmj.c181.long for guidelines on how to de-identify and prepare clinical data for publication. For a list of recommended repositories, please see https://journals.plos.org/plosone/s/recommended-repositories. You also have the option of uploading the data as Supporting Information files, but we would recommend depositing data directly to a data repository if possible.

Please update your Data Availability statement in the submission form accordingly.

**Answer:** We have updated our Data Availability Statement as follows:

The corresponding author has full access to the original dataset supporting this article. In accordance with the trial’s ethical approval and participant consent procedures, data will only be available ‘on request’ and is subject to approval by the Trial Steering Committee, a supplementary approval by the University of Toulouse III Ethics Committee and a data sharing agreement. Please contact either the corresponding author or the trial manager Dr Niamh M Redmond ([niamh-maria.redmond@univ-tlse3.fr](mailto:niamh-maria.redmond@univ-tlse3.fr)) for further information.

**5. In the online submission form, you indicated that [Requests for further information can be directed to the corresponding author.]**

All PLOS journals now require all data underlying the findings described in their manuscript to be freely available to other researchers, either 1. In a public repository, 2. Within the manuscript itself, or 3. Uploaded as supplementary information.

This policy applies to all data except where public deposition would breach compliance with the protocol approved by your research ethics board. If your data cannot be made publicly available for ethical or legal reasons (e.g., public availability would compromise patient privacy), please explain your reasons on resubmission and your exemption request will be escalated for approval.

**Answer:** Our Data Availability Statement clarifies the restrictions we are required to comply with regard to data sharing: The corresponding author has full access to the original dataset supporting this article. In accordance with the Trial’s Ethical approval and participant consent procedures, data will only be available ‘on request’ and is subject to approval by the Trial Steering Committee, a supplementary approval by the University of Toulouse III Ethics Committee and a data sharing agreement. Please contact either the corresponding author or the trial manager Dr Niamh M Redmond ([niamh-maria.redmond@univ-tlse3.fr](mailto:niamh-maria.redmond@univ-tlse3.fr)) for further information.

**6. Please include your full ethics statement in the ‘Methods’ section of your manuscript file.** In your statement, please include the full name of the IRB or ethics committee who approved or waived your study, as well as whether or not you obtained informed written or verbal consent. If consent was waived for your study, please include this information in your statement as well.

**Answer:** The full ethics statement is now included in the ‘Method’ section as follows:

“Ethical approval for the study 2021-349 was received from the local research ethics committee, the CER (comité d'éthique de la recherche) at the University of Toulouse III Paul Sabatier, France (ref 2021–349, dated 8th March 2021, category MR004) and the DECODE trial was registered on the 17th of November 2020 on ClinicalTrials.gov with the trial registration number 2020-A01687-32. Informed written consent for participation in the trial and the qualitative studies was obtained from all participating general practitioners.”

**7. Please include captions for your Supporting Information files at the end of your manuscript, and update any in-text citations to match accordingly.** Please see our Supporting Information guidelines for more information: http://journals.plos.org/plosone/s/supporting-information.

[Note: HTML markup is below. Please do not edit.]

**Answer:** A section titled “Supporting information” was included at the end of the manuscript, listing supporting information captions. The supporting information files at the end of the manuscript has now been reviewed and updated to match accordingly.

Reviewers' comments:

**Reviewer #1:**

**1.** Thank you for the opportunity to review this manuscript, which addresses an important field for research, namely how health literacy is vital for participation in CRC screening. However, the manuscript has several elements that it addresses. While the introduction concerns CRC screening, the study that has been conducted is about how GPs evaluate a learning intervention about health literacy and CRC screening. It is a bit confusing that the introduction is more about the umbrella study, rather than the issue at stake in this particular qualitative study.

**Answer:** Thank you for the feedback. To clarify the research question and the objective of the study, we have replaced lines 102–110 (from 'To add to the body of knowledge > widespread implementation' in the introduction section) with the following sentence:

“To this end, we developed and conducted an e-learning training program on HL and CRC screening for GPs practising in deprived areas across four regions of France. This training program was part of a multicentre, cluster-randomised controlled trial (REF 30) which aimed to increase CRC screening uptake and also included a patient-facing intervention (pictorial brochure and video) conducted during consultations. In this trial, we included several qualitative studies to gain a deeper understanding of the integration of the intervention among both targeted patients and participating GPs.

Here, we present a nested qualitative study with GPs in the intervention group. The objective of this study was to evaluate the acceptability of an e-learning training programme on HL and CRC screening for GPs practising in deprived areas. We aimed to assess whether this training could be implemented among a broader population of GPs and explore strategies to promote its widespread implementation across France and within the healthcare system”.

**2. A second overall comment is about the choice of qualitative interviews** and how the data from the interviews is presented in the results section. While the idea of doing a qualitative interview study is good, it is not clear from the results what this choice of method has contributed to, which could not have been identified through using a survey with open ended questions. I would urge the authors to clarify why this design was chosen and what its contribution is. Perhaps this manuscript would benefit from being presented as an evaluation study rather than a qualitative study?

**Answer:** We chose a qualitative study because it is a recognized and validated method for assessing the acceptability and implementation potential of an intervention (<https://pmc.ncbi.nlm.nih.gov/articles/PMC3758883/>). Qualitative studies nested with a randomised controlled trial (RCT) are common in public health research and add complementary value to the often purely quantitative aspect of the RCT. This qualitative approach was used to complement the quantitative data collected to assess the acceptability of the training.

In the design section, we included the following in order to provide details on the choice of method and included the reference above:

“We used semi-structured interviews to enable a more detailed analysis and a richer insight into the experience of the e-learning training among GPs in the intervention arm. This qualitative approach allows for a more in-depth and flexible exploration of stakeholders' perspectives compared to a survey or other quantitative methods. It also enables researchers to probe deeper into specific topics or questions. In particular, GPs could directly suggest ways to improve the training in terms of structure, format, content, and methods of dissemination to facilitate its future implementation”.

**Some more detailed comments:**

**3. 104-107: The introduction is very well written. However, instead of talking about the number of qualitative studies nested within your RCT, could you rather explain why you do the study presented here? What was the current study expected to contribute to the overall study?**

**Answer:** Thank you for your positive feedback on the introduction. We have refined the study’s objective and clarified its intended broader contribution as follows at the end of the introduction section:

“The objective of this study was to evaluate the acceptability of an e-learning training programme on HL and CRC screening for GPs practising in deprived areas. We aimed to assess whether this training could be implemented among a broader population of GPs and explore strategies for promoting its widespread implementation across France and within the healthcare system”.

**4. 115-116: “We reported our results using the Consolidated Criteria for Reporting Qualitative Research (COREQ) (S1. Appendix COREQ checklist)”**

**COREQ is a list of issues that needs to be addressed in each article. However, it is not sufficient to create a high-quality article. I would suggest that the authors of this article look at more qualitative articles to see how they bring depth to their findings. Also, I would suggest reading Sarah Tracy (2010) article on “Big 8 criteria for qualitative research”.**

**Answer:** We ensured that our study adhered to the highest standards of qualitative research by designing a robust qualitative study nested within the trial. Our team includes several researchers with over 15 years of experience conducting qualitative studies, ensuring methodological rigour and expertise.

The use of the COREQ checklist is widely used in qualitative research and is listed as a recommended checklist for research quality and transparency in qualitative studies on the Equator network ; <https://www.equator-network.org/reporting-guidelines/coreq/>.

The COREQ checklist was first published in 2007 (<https://doi.org/10.1093/intqhc/mzm042>) and, in addition, PLOS recommends its use and inclusion for all submitted qualitative studies for review : <https://journals.plos.org/plosone/s/submission-guidelines#loc-qualitative-research>.

Thank you for the recommended reference to the “Big 8 criteria for qualitative research”. After reviewing the eight criteria of this pedagogical model, we found that our study aligns well with the key markers of quality. Therefore, we are including this reference.

"We also adhered to the 'Eight Big-Tent Criteria for Excellent Qualitative Research' (<https://journals.sagepub.com/doi/10.1177/1077800410383121>) a flexible pedagogical model that distinguishes between means (methods and practices) and outcomes (ends). It introduces eight key markers of quality: (a) worthy topic, (b) rich rigour, (c) sincerity, (d) credibility, (e) resonance, (f) significant contribution, (g) ethics, and (h) meaningful coherence."

**5. Something looks strange in the document here:**

**147 Setting and participants of the qualitative study**

**148 Recruitment of GPs to the qualitative study**

**Answer:** "Thank you for your observation. For clarity, we deleted the Level 2 heading 'Recruitment of GPs to the qualitative study' and kept only 'Setting and participants of the qualitative study.'

**6. 150: what is the purpose of including gender of GP as a factor for the sample? Do you have literature suggesting that gender would be important for the theme of the study?**

**Answer:** Although we identified several studies (<https://pmc.ncbi.nlm.nih.gov/articles/PMC6731795/> <https://pubmed.ncbi.nlm.nih.gov/8332153/> https://pubmed.ncbi.nlm.nih.gov/26765146/) showing that GP gender was independently associated with the quality of care regarding process indicators for cancer screening, we did not investigate this aspect due to the small sample size. We reported gender as well as age as basic socio-demographic information on the sample recruited.

**7. 154: How did you evaluate that 20 participants were necessary for saturation? What kind of saturation are you referring to here?**

**Answer:** First of all, we were limited by the number of GPs participating in the DECODE trial. We contacted all 32 GPS in the intervention group, including those that had withdrawn from the study but had completed the training, and invited them to participate in interviews. Based on our previous experience of qualitative studies of this type, we expected roughly 20 participants to reach saturation.

We have revised the sentence as follows and included a reference:

“Based on previous experience of qualitative studies of this type, we expected roughly 20 participants to reach thematic saturation (<https://journals.sagepub.com/doi/10.1177/1525822X05279903>). To ensure a balance across regional centres we completed 22 GP interviews (4-5 participants per region)

**8.** **158: what is meant by “implementation potential”? Is this the potential for implementing the GP training among GPs? Or is it about implementation of HL knowledge among patients?**

**Answer:** We apologise for this confusion. We meant the potential for implementing the training among the broader population of GPs in other areas of France. The clarification has been added throughout the manuscript

**9. Data analysis: was the analysis inspired by any analytical approach? Which literature did you use when deciding on inductive thematic analysis? Could you explain more in detail how you used NPT during your analysis?**

**Answer:** Data were analysed thematically, using the Framework Approach. We based our decision to use inductive thematic analysis on key methodological literature that emphasises its strengths in allowing themes to emerge directly from the data without being constrained by preconceived structures. The following literature was used:

**Refs**

<https://doi.org/10.1177/1609406917733847>

<https://www.bmj.com/content/381/bmj-2022-074256>

We used the NPT model as it was well-suited to our objective of assessing the acceptability of training and the implementation of its strategies, while also identifying recommendations for the sustainability of future training and clinical practices. This framework was also used in similar studies, relating to training initiatives, share decision-making or decision aids.

**Refs**

https://pubmed.ncbi.nlm.nih.gov/25917169/

<https://pubmed.ncbi.nlm.nih.gov/38192729/>

https://implementationscience.biomedcentral.com/articles/10.1186/1748-5908-8-102

<https://pmc.ncbi.nlm.nih.gov/articles/PMC8108365/#Bib1>

For clarity, the data analysis section has been revised as follows:

“A framework analysis with inductive methods (**Refs x2 above)** was initially conducted to identify any emerging codes. NPT was then applied as an analytical lens to examine the data, as it provides a suitable framework for exploring participants' views on how interventions impact their work and can be integrated into routine clinical practice. This framework has been used in studies on training initiatives, shared decision-making, and conversation aids (**Refs x4 above**). This theory aligned well with our objective of assessing the acceptability of the training while identifying recommendations for the sustainability of future training and practices. The coding process was guided by the core constructs and components of NPT”

**10. 181: I would suggest deleting this sentence, it is unnecessary to explain this.**

**Answer:** We agreed and deleted the sentence.

**11. 187: Characteristics of participants should be moved to the methods section.**

**Answer:** We believe that the participant characteristics should remain in the 'Results' section, as they represent the 22 GPs who actually participated in an interview out of the 32 GPs initially contacted. Among these, 10 declined for various reasons described in the manuscript.

**12. Table 3. Summary of major themes according to NPT constructs is difficult to grasp due to the explaining sentences being very long, complex and using many concepts.**

**Answer:** Table 3 was reviewed and has been shortened for clarity, as follows:

| **NPT construct** | **Major themes** |
| --- | --- |
| Sense-making | GPs felt the training would be helpful for better communication with patients with low HL. It also helped with CRC screening, a common but challenging topic in preventive care. |
| Cognitive participation | GPs found the training format engaging. Interest in HL was another key driver of participation. |
| Action | GPs considered virtual group sessions essential but recommended they be more grounded in real-life situations. |
| Monitoring | GPs recommended expanding the training to more GPs, provided it stays brief, more interactive, and focused on HL. |

**13. 210: t factor?**

**Answer:** Thank you for your observation. It was a typo that has now been corrected.

**14. 221: you write that “A few respondents (n=3)”. What is the purpose of counting how many said what in a qualitative study? This is also done in line 229 (and several places in the results section) where it says that “The majority of GPs (17 out of 22)…”. This makes me question if the results are analyzed through a qualitative approach, or if this is rather an evaluation of the HL training. The results are solely descriptive, even when using the theoretical framework of NPT. This is a weakness of the study, since the analysis does not utilize the qualitative aspect of the data.**

**Answer:** The number of respondents was reported for accuracy purposes, to faithfully reflect the prevailing trends (whether consensus or divergent views) and to develop recommendations for improving the training and, more generally, for better raising GPs' awareness of health literacy.

The physicians' responses regarding aspects of the training were inherently more descriptive than analytical, often emphasizing practical application, in line with the objective of our study to assess the acceptability of the training. As previously noted, (point 2), the qualitative approach was used to complement the quantitative data collected to assess the acceptability of the training, providing deeper insights than a questionnaire. We believe that the depth of reflection demonstrated by GPs across the four constructs of the NPT, particularly their internal motivations to learn about HL, and their readiness to implement positive changes at both individual and clinic-level practice, goes beyond a simple evaluation of a training. It reflects a genuine reflective process, driven by their ability to sincerely question their own practices and embed improved communication approaches into routine care, extending beyond this trial and the specific issue of screening.

**Reviewer #2: Positive Aspects:**

1. The study tackles the low CRC screening rates, especially in deprived areas, and emphasizes the role of health literacy, which is crucial for improving public health outcomes.

2. The qualitative study offers deep insights into GP perspectives, providing valuable input for future interventions.

3. Focusing on GP training is strategic, as they are key influencers in promoting CRC screening.

4. The paper offers practical suggestions for improving training programs, which could aid in scaling up such initiatives.

Overall, the manuscript provides important insights into GP training's role in promoting CRC screening and health literacy. With some additional discussion on the points raised, the study could significantly contribute to the field.

We thank Reviewer #2 for their positive feedback.

**Suggestions for Improvement:**

**1. The 68% attendance in booster sessions and participants' fading memories suggest the need for repeated reinforcement training. Could the authors discuss the necessity of such sessions and how future training could be enhanced to ensure lasting effects?**

**Answer:** Thank you for your suggestion. To clarify, the Booster training sessions were carried out to remind GPs about recruitment of patients into the trial, the intervention and how to communicate with patients about the CRC screening test using the training they were initially provided with, approximately 6 months after the start of recruitment. For some GPs, the qualitative interviews were done 9 months to a year beyond that.

Reasons for not attending Booster training sessions were lack of time on behalf of a few GPs, however there was also a logistical issue for other GPs, with regards to a prolonged ‘extension to funding’ request, which, when granted, rendered the Booster training obsolete, because there was little time available to implement the additional training in the time that remained of the recruitment period. We hope this goes some way to contextualising the low attendance rate of the Booster training sessions.

Due to word limit restrictions, we don’t feel it is necessary to explain this precise detail in the article but we have included the following recommendations in the conclusion section to suggest strategies for sustaining the lessons from the training over time:

“To sustain the lasting impact of the training, we recommend annual online refresher sessions to reinforce learning and keep skills up-to-date. These sessions should include interactive peer discussion groups for sharing experiences and addressing challenges through case studies. Additionally, brief, asynchronous micro-learning modules could be developed, allowing GPs to review key concepts at their convenience. Finally, newsletters and distance-learning need assessment surveys should be used to tailor content to GPs' evolving needs, supporting ongoing self-directed learning.”

**2. Further analysis on the relationship between health literacy and CRC screening uptake could be beneficial. Are there specific health literacy aspects that significantly influence screening behavior, and how can they be targeted in training?**

**Answer:** Thank you for this suggestion and the interesting question posed. The trial itself was based on a previous successful randomised controlled trial in the United States conducted in veterans in 2005 (Ferreira et al. <https://doi.org/10.1200/JCO.2005.07.049>). Our aim was to determine if a similar complex intervention (HL training and CRC screening information tools for patients) could be replicated in a French context and moreover within deprived areas with the goal of reducing health and social inequalities.

A number of skills are required by any one person in any specific health context, to obtain health information (or health services), understand this information and act to be able to make appropriate decisions about their health. In addition, the influences of education, culture, upbringing and previous experience can also impact a person's ability to engage with health prevention services, like screening. Therefore, due to the contextual and multi-skill aspect, it is difficult to determine if any one element of health literacy may significantly increase screening. (<https://pubmed.ncbi.nlm.nih.gov/24207115/>)

Each component of health literacy or combination of components may influence a person’s ability to make a decision about participating in cancer screening. This is why we tested a mixed, complex intervention already proven in one context (Ferreira et al, 2005), and conducted a similar RCT in a different context in France (specifically targeting GP practices in low socio-economic areas where the CRC screening participation rates are often lower than the national average and low health literacy among the population is often more common). Our nested qualitative study to evaluate the acceptability of the e-learning training programme with GPs provides valuable information on the training aspect and recommendations on how it could be improved.

Drawing on the Sorensen model (https://pmc.ncbi.nlm.nih.gov/articles/PMC3292515/) we believe that the overall intervention, including the training, contributed to improving patients’ understanding, clarified the personal relevance of screening benefits, provided practical information to facilitate its application and therefore increase motivation to through discussions with GPs.

We have added further information and references into the discussion section as follows:

“Placing an even greater emphasis on HL and communication strategies was deemed a top priority by participating GPs as observed in another study conducted in the French context (https://pubmed.ncbi.nlm.nih.gov/35724234/), which underscored the importance of promoting patient-physician dialogue through clear and motivating explanations of the screening procedure. However, while training GPs in cancer risk communication can improve their counselling behaviours, it does not always translate into higher screening rates among patients with limited HL (<https://pubmed.ncbi.nlm.nih.gov/24590734/>).

“Receiving feedback about their screening rates was viewed as encouraging promotion of screening, consistent with findings from several reviews that demonstrated increased uptake as a result of this intervention. (54–56). Provider feedback interventions have also proven effective in increasing breast and cervical screening uptake <https://pubmed.ncbi.nlm.nih.gov/33003999/> <https://pubmed.ncbi.nlm.nih.gov/24590734/>).

“However, there is insufficient evidence to assess the effectiveness of provider incentives in increasing screening rates for breast, cervical, or colorectal cancers (<https://pubmed.ncbi.nlm.nih.gov/18541190/>)

**3. More context on the current CRC screening situation in the study area, including any identified initiatives or barriers, would provide a clearer backdrop for the training program.**

**Answer:** Thank you for your suggestion. In line with the suggestion by review 1 to reduce the information on CRC and screening and with the word limit, we have attempted to strike a balance and keep this aspect brief, although it is an important context for this study. The second paragraph had already briefly summarised the context in France with regard to the national organised colorectal cancer screening programme, as follows:

“The French national CRC-screening programme was launched in 2009 and targets people aged 50 to 74. Eligible persons receive standardised invitation letters, every 2 years, to visit their general practitioner (GP), specialist or pharmacist to obtain a home and self-administered FIT test (4). However, CRC screening test uptake remained suboptimal at 34.3% in 2021-22 and far below European targets of between 45% (minimum) and 65% (desirable) (5,6).”

We have modified this paragraph as follows:

“The French national CRC-screening programme was launched in 2009 and targets people aged 50 to 74. Eligible persons receive standardised invitation letters, every 2 years, to visit their general practitioner (GP), specialist or pharmacist to obtain a home and self-administered FIT test (4). However, CRC screening test uptake remained suboptimal at 34.3% in 2021-22 and far below European targets of between 45% (minimum) and 65% (desirable) (5,6). Screening uptake in France is socially graded (<https://pubmed.ncbi.nlm.nih.gov/19740776/>, <https://pubmed.ncbi.nlm.nih.gov/22704817/>) decreases with increasing levels of deprivation and is lower among those in socially deprived areas <https://pubmed.ncbi.nlm.nih.gov/19740776/>, <https://pubmed.ncbi.nlm.nih.gov/20403737/>)

Similar to global trends….

“There is some evidence that patient navigators can facilitate participation in CRC screening in low socio-economic areas (<https://pubmed.ncbi.nlm.nih.gov/36609248/>) but these could be costly in terms of both time and financial costs”.
